# Effect of Dryland-to-Paddy Conversion on Soil Aggregate Phosphorus Fractions and Microbial Functional Diversity in a Typical Black Soil Region of the Sanjiang Plain

**DOI:** 10.3390/microorganisms14030658

**Published:** 2026-03-14

**Authors:** Bo Bo, Xinghong Liu, Zijian Xie, Chunhua Li, Yang Wang, Chun Ye

**Affiliations:** 1 National Engineering Laboratory for Lake Pollution Control and Ecological Restoration, Chinese Research Academy of Environmental Sciences, Beijing 100012, China; bobo.boshi@163.com (B.B.);; 2 Key Laboratory for Lake Pollution Control of the Ministry of Ecology and Environment, Chinese Research Academy of Environmental Sciences, Beijing 100012, China; 3 College of Environmental and Safety Engineering, Shenyang University of Chemical Technology, Shenyang 110142, China

**Keywords:** dryland-to-paddy conversion, phosphate-solubilizing microorganism, phosphorus fractions, soil aggregates, black soil region

## Abstract

The Sanjiang Plain is a key black soil agricultural zone in Northeast China. The conversion of dry-lands (DL) to paddy fields (PF) alters soil aggregate phosphorus (P) fractions and microbial diversity, yet the underlying mechanisms are unclear. This study compared DL and PF (converted from DL) soils. The results showed that electrical conductivity (EC) and soil organic carbon (SOC) increased significantly after the dryland-to-paddy conversion (*p* < 0.05). The proportions of macroaggregates and microaggregates increased, while the silt+clay fraction declined (*p* < 0.05), indicating enhanced aggregate stability. Soil total P increased by 16.04%, of which 83.81%, was attributed to macroaggregate-associated P. The dominant P fractions shifted from NaOH-Po to NaOH-Pi and HCl-Pi. The land-use change also markedly altered the soil microbial community structure, leading to increased abundances of *Bradyrhizobium* and *Pseudomonas* and decreased abundances of *Streptomyces* and *Mesorhizobium*, collectively driving the transformation of P fractions. The key functional genes identified were *gcd*, *phoD*, and *phnA*. However, this study did not capture the temporal dynamics of P forms and microbial community structure across different stages of land-use conversion. Future research should track these dynamics throughout the conversion process to clarify the mechanisms of P evolution.

## 1. Introduction

Soil phosphorus (P) is an essential nutrient for plant growth and development, playing a key role in soil nutrient cycling [1,2]. In soil, P primarily exists in inorganic forms, such as calcium-bound and iron/aluminum-bound phosphates, as well as in organic forms, such as phytate and nucleic acids [1]. Soil aggregates regulate non-bioavailable P primarily through physical protection, particularly within microaggregates [3]. As the basic unit of soil structure, soil aggregate are the primary sites for the decomposition and transformation of soil organic matter (SOM), thereby significantly influencing soil nutrient accumulation and transformation [3,4]. Stable aggregates enhance soil resistance to water flow and weathering of erosion, thereby reducing nutrient loss caused by surface runoff [5]. Aggregates can store more total phosphorus (TP), which slows down the decomposition and mineralization of SOM, thus reducing P availability [6]. The distribution of P components within soil aggregates is influenced by aggregate size, SOM, and pH [7]. Additionally, aggregates of different particle sizes exhibit varying capacities for P adsorption and release. Small aggregates generally possess a stronger P adsorption capacity due to their larger specific surface area, whereas macroaggregates may limit nutrient availability [7,8]. Therefore, it is important to study the correlation between soil aggregates and P to understand the P cycle in soil at the microscale.

Studying soil P fractions is important for understanding P migration and transformation, and for evaluating P bioavailability [9]. Soil TP is often a poor indicator of its availability and transformation pathways [10,11]. The Hedley sequential extraction method offers insights into the availability and stability of P forms by assessing the binding strength of P within the soil matrix [11,12]. Generally, P fractions extracted by the Hedley sequential extraction procedure can be categorized into three types: highly active P, moderately active P, and lowly active P [11,13]. Highly active P, regarded as the most bioavailable P component, mainly consists of H_2_O-P, NaHCO_3_-Pi, and NaHCO_3_-Po [11,13]. Moderately active P, which includes NaOH-Pi and NaOH-Po, is usually combined with iron and aluminum oxides and is prone to combine with calcium; it exhibits potential availability for plant uptake and can be released under specific soil conditions [11,13]. Lowly active P, including HCl-Pi, conc.HCl-Pi, conc.HCl-Po and Residual-P, remains relatively stable in the soil matrix and has a slow but prolonged P release potential [13]. Thus, exploring the characteristics of P fractions in soil helps to clarify the key processes involved in the transformation of P components and supports effective agricultural P management.

Microorganisms play a vital role in the soil P cycle and are considered key drivers of soil P turnover [13,14]. Phosphate-solubilizing microorganisms (PSMs) can enhance the soil P availability by promoting the desorption of inorganic P or the decomposition of organic P. PSMs are found among bacteria, actinomycetes, and fungi [11,14]. Acid hydrolysis is the primary mechanism by which PSMs solubilize P. Organic acids produced by PSMs can block P adsorption sites in the soil or form complexes with cations on the soil mineral surfaces, thereby increasing the P availability [11,13]. In addition, PSMs can mineralize organophosphate by secreting acid or alkaline phosphatases, converting it into soluble P that plants can absorb and utilize [14,15]. For instance, *Brevundimonas* and *Paenibacillus* can simultaneously dissolve inorganic P (e.g., calcium phosphate) and mineralize organic P (e.g., phosphates); the phosphatases they secrete hydrolyzes the organic P bonds, releasing phosphate ions available for plant uptake [16]. Additionally, PSMs release CO_2_ and other compounds through respiration, lowering the pH of the surrounding soil environment and thereby facilitating the dissolution of insoluble phosphates [13,17]. Consequently, investigating the structure of soil microbial communities and their interactions with P fractions is essential for elucidating the key regulatory mechanisms through which phosphate-solubilizing microbial groups activate soil P.

Xingkai Lake, situated on the Sino-Russian border in southeastern Heilongjiang Province, is the largest international boundary lake in northeast Asia [12,18]. The region is characterized primarily by black soil and is the core grain-producing area of the Sanjiang Plain [19]. In recent years, the area of farmland in the Xingkai Lake Basin has been continuously increasing. Paddy fields (PF) have increased significantly and have been converted mainly from dry-lands (DL) [12]. The land use conversion from DL to PF involves transforming low-lying, flood-prone and low-yielding DL into high-yield PF, thereby enhancing the soil productivity [20]. This conversion also alters soil moisture content, redox potential, pH, SOM, and microbial community structure, thereby influencing soil P transformation [21,22]. Therefore, exploring changes in P fractions and microbial communities is important to understand the mechanisms that drive the soil P cycle during the dryland-to-paddy conversion.

This study was conducted in a black soil region where DL had been converted to PF. Previous studies have shown that the dryland-to-paddy conversion alters soil physicochemical properties, resulting in the redistribution of P among different aggregate components. Such environmental changes can have an impact on the composition of soil microbial communities. However, the correlation between the dynamics of P fractions and both PSMs as well as phosphorus-solubilizing functional genes remains unclear. We hypothesize that the dryland-to-paddy conversion will have an impact on the structure of soil aggregates and the P components, thereby influencing the composition of the microbial community in the soil and the expression of phosphorus-solubilizing functional genes. The main objectives of this study were: (1) to investigate the structure of soil aggregates and the characteristics of P fractions; (2) to elucidate the soil microbial community structure and key functional genes involved in P cycling; (3) to determine the relationship between soil P fractions and microbial communities. The results are expected to provide scientific support for improving soil P management in the black soil regions of Northeast China.

## 2. Materials and Methods

### 2.1. The Study Area

**Figure 1:**
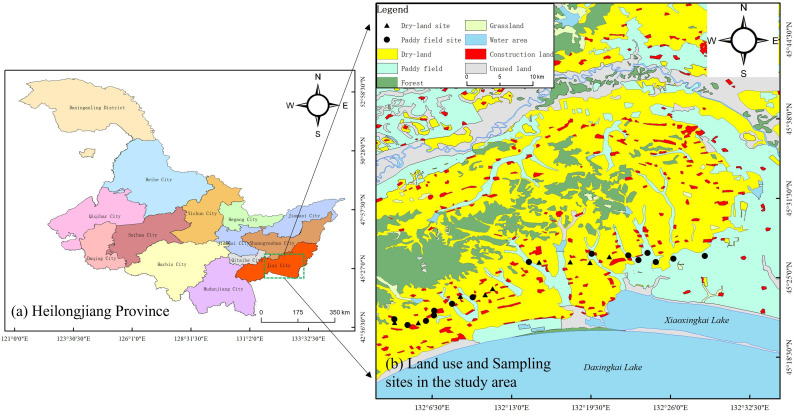
Land use and sampling sites in the study area.

The study area is located in Jixi City, Heilongjiang Province, and is characterized primarily by plains. According to the Köppen–Geiger climate classification system, this region is classified as type Dwc, characterized by a subarctic climate with dry winters and humid summers [23]. The annual precipitation is 559 mm, with approximately 70% of the rainfall concentrated between June and September [12]. Land use in this region has changed significantly. From 1990 to 2020, the area of PF in the Xingkai Lake Basin increased by nearly 688 km^2^, of which 520 km^2^ were converted from DL, accounting for about 30% of the total PF area (Figure 1) [12,24].

### 2.2. Sample Collection and Processing

The sampling area, which included both DL and PF sites, was located within the agricultural area of the Xingkai Lake Basin on the Sanjiang Plain (132°6′–132°26′ E, 45°18′–45°31′ N). Soil samples were sampled in November 2024 after the harvest season. A total of 24 sampling points were established, including 9 sites in the DL area and 15 sites in the PF area (Figure 1). The land-use conversion from DL to PF had taken place more than five years ago. All sampling points were located in the coastal area of Xingkai Lake with similar background geological conditions.

At each sampling point, three samples were collected and then mixed to form one composite sample. A soil auger was used to collect samples from the top-layer (0–20 cm). Surface litter was carefully removed before soil collection. Each composite sample was divided into three parts, immediately stored in a portable refrigerator, and promptly transported to the laboratory. The first part was used for microbiological testing. The remaining samples were then air-dried until a constant weight was achieved. After drying, the second part was ground and passed through sieves of 2 mm (10-mesh), 0.25 mm (60-mesh), and 0.15 mm (100-mesh) to analyze basic physical and chemical properties [25].

The third part of each sample was separated into three aggregate fractions by using the wet-sieving method: silt+clay (<0.053 mm), microaggregates (0.053~0.25 mm), and macroaggregates (0.25~2 mm) [11,26,27]. First, 100 g of air-dried soil (pre-sieved through 2 mm) was capillary-wetted and passed through a 0.25 mm sieve; the soil retained on the sieve was classified as macroaggregates. Second, the remaining soil was sieved through a 0.053 mm sieve, the soil retained on the sieve was microaggregates. Third, the remaining soil solution was centrifuged at 2500 rpm for 10 min, and the resulting pellet was silt+clay.

### 2.3. Determination of Basic Soil Properties

Soil pH and electrical conductivity (EC) were measured using pH and EC meters with a soil-to-water ratio of 1:2.5. Soil organic carbon (SOC) was determined by the dichromate digestion method [13,25]. Total nitrogen (TN) was measured using the Kjeldahl method [13,25]. TP was digested with HNO_3_-HF using microwave digestion and analyzed by ICP-OES (Optima 5300DV, PerkinElmer, Waltham, MA, USA) [13,25]. The particle size distribution of soil aggregates was analyzed with a laser diffraction particle size analyzer (Mastersizer 2000, Malvern Panalytical Ltd., Malvern, UK).

### 2.4. Analysis of Soil Phosphorus Fractions

Soil P fractions were obtained using the Hedley sequential extraction method [11,13]. This procedure separates nine P components: H_2_O-Pi, NaHCO_3_-Pi, NaHCO_3_-Po, NaOH-Pi, NaOH-Po, HCl-Pi, conc.HCl-Pi, conc.HCl-Po and Residual-P [13]. The inorganic P (Pi) content in each extract was measured by the molybdenum colorimetry method. TP (Pt) in the filtrates was measured by the molybdenum colorimetry method after digestion with 1 mL of HClO_4_ and H_2_O_2_ solution [11]. Organic P (Po) was calculated as the difference between TP and Pi (Po = Pt − Pi).

The sequential extraction steps were conducted as follows: (1) H_2_O-Pi, 0.5 g soil was shaken with 30 mL of deionized water for 16 h; (2) NaHCO_3_-P, the remaining soil was extracted with 30 mL of 0.5 M NaHCO_3_ solution for 16 h; (3) NaOH-P, the remaining soil was shaken with 30 mL of 0.1 M NaOH solution for 16 h; (4) HCl-Pi, the remaining soil was extracted with 30 mL of 0.5 M HCl solution for 16 h; (5) conc.HCl-P, the remaining soil was treated with 10 mL of 1 M HCl solution and heated in an 80 °C water bath for 10 min, then was shaken with 5 mL 0.5 M HCl solution for 16 h; (6) Residual-P, the remaining soil was digested using 5 mL of H_2_SO_4_ and H_2_O_2_ [11,13].

### 2.5. High-Throughput Sequencing and Bioinformatic Analysis of Soil Microorganisms

Metagenomic sequencing and subsequent analyses were performed by Wekemo Tech Group Co., Ltd. (Shenzhen, China). For each sample, three independent biological replicates were analyzed. Genomic DNA was extracted from 0.25 g soil using the cetyltrimethylammonium bromide (CTAB) method [28,29,30]. Briefly, samples were lysed in CTAB buffer with lysozyme at 65 °C, followed by sequential phenol–chloroform extraction and isopropanol precipitation. DNA pellets were washed with ethanol, dissolved in ddH_2_O, and treated with RNase A [26,27,28]. DNA quality and concentration were assessed using a Fragment Analyzer (5400, Agilent, Santa Clara, CA, USA), with sample preparation performed using a benchtop centrifuge (Fresco 17, Thermo, Waltham, MA, USA) and vortex mixer (ThermoMixer C, Thermo, Waltham, MA, USA) [29,30].

Sequencing libraries were constructed with the NEBNext Ultra II DNA Library Prep Kit (Illumina, San Diego, CA, USA) using an automated liquid handler (Biomek i7 Hybrid, Beckman Coulter, Brea, CA, USA), with fragmentation performed using an ultrasonicator (LE220R-plus, Covaris, Woburn, MA, USA), size selection performed using the AMPure XP system (Beckman Coulter, Brea, CA, USA), and amplification performed using a PCR thermal cycler (T100, BIO-RAD, Hercules, CA, USA). Library quality and concentration were verified using the Fragment Analyzer (5400, Agilent, Santa Clara, CA, USA), a touch q-PCR system (BIO-RAD, Hercules, CA, USA), and a fluorometer (Qubit 3.0, Invitrogen, Carlsbad, CA, USA). Library pooling was performed using an automated pooling system (Echo 550, Labcyte, San Jose, CA, USA). Sequencing was conducted on a sequencing platform (NovaSeq Xplus, Illumina, San Diego, CA, USA). Raw reads were processed using Trimmomatic (v0.39) with parameters: ILLUMINACLIP:adapters_path:2:30:10 SLIDINGWINDOW:4:20 MINLEN:50, removing adapters and low-quality bases (Q < 20). Host DNA sequences were removed by alignment with Bowtie2.

Taxonomic profiling was performed with Kraken2 against a customized database (updated October 2024) containing bacterial, fungal, archaeal, and viral sequences from the NCBINT and RefSeq databases, with the inclusion criterion that the microorganisms had complete genome assemblies. Species abundance was estimated using Bracken.

Genes related to P metabolism and cycling were identified by aligning high-quality reads against the UniRef90 protein database using HUMAnN3, followed by functional annotation against the KEGG orthology (KO) database. Their abundance was determined by summing the normalized abundances of UniRef90 gene families corresponding to KEGG orthologs involved in P cycling [13].

### 2.6. Data Analysis

The Wekemo Bioincloud Platform was used to extract and analyze microbial abundance and functional genes, and to generate clustered heatmaps based on one-way ANOVA analysis [31]. OriginPro 2022 V9 was employed to visualize the composition of P fractions, create bar charts of the phosphorus-solubilizing functional genes, and show the abundance of PSMs [32]. RStudio 9.5.191.52 was used to construct a structural equation model (SEM) using the PLS-PM path model to analyze the relationships among soil properties, P-cycling functional microorganisms, functional gene abundance, and P content in aggregates [33].

## 3. Results

### 3.1. Effects of Land-Use Change on Basic Soil Properties

The soil in the study area was moderately acid. After the conversion from DL to PF, soil EC and SOC increased significantly (*p* < 0.05), while pH, TN and TP showed an increasing trend (Table 1). Moreover, the proportion of microaggregates and macroaggregates in PF was significantly higher than those in DL (*p* < 0.05; Table 1). In contrast, the proportion of the silt+clay fraction in DL was significantly higher than that in PF (*p* < 0.05; Table 1). The stability of the aggregates tended to increase after the conversion.

**Figure 2:**
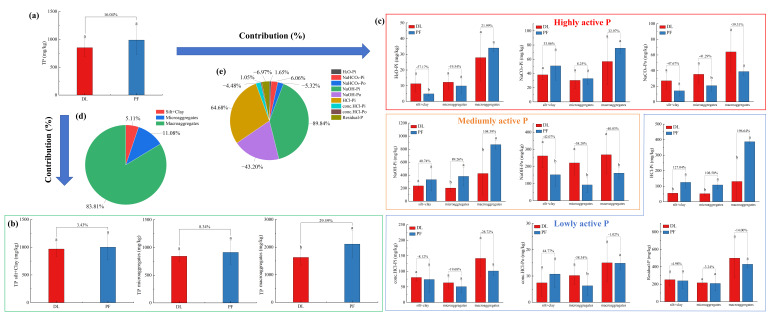
Content of phosphorus fractions in soil aggregates under dry-lands (DL) and paddy fields (PF) (**a**) Total phosphorus content; (**b**) total phosphorus content of soil aggregates; (**c**) content of different phosphorus fractions in each soil aggregate; (**d**,**e**) contribution of aggregates and phosphorus fractions to the variation in phosphorus content. Significant differences between DL and PF are denoted by different lowercase letters (*p* < 0.05).

**Figure 3:**
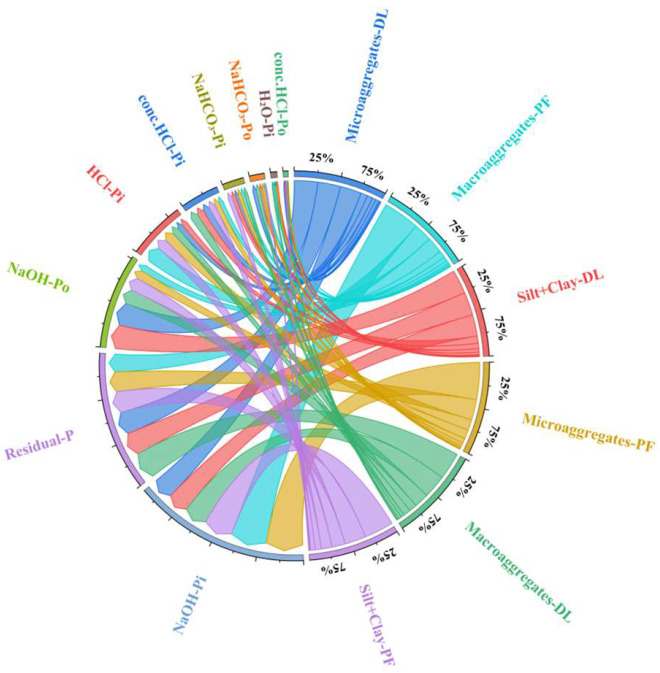
Proportional distribution of phosphorus fractions in soil aggregates from dry-lands (DL)and paddy fields (PF).

The soil in the study area was classified as the levels of rich and relatively rich of TP (Table S1) [19]. This soil exhibited pronounced P accumulation in the topsoil, distinct P fractions, a highly concentrated distribution pattern within the profile, and certain associated environmental risks [9,20]. Compared to DL, the soil TP in PF increased by 16.04%. In DL, the predominant P fractions were NaOH-Pi, NaOH-Po and Residual-P, accounting for 23.98–26.12%, 16.47–27.03% and 25.71–30.66%, respectively (Figure 2). In PF, the main components were NaOH-Pi, HCl-Pi and Residual-P, constituting 33.07–41.89%, 11.84–18.32% and 20.35–23.92%, respectively (Figure 2 and Figure 3). After the dryland-to-paddy conversion, the P composition shifted from being dominated by NaOH-Po to being dominated by NaOH-Pi and HCl-Pi. Specifically, NaOH-Pi increased by 8.76–17.91% and HCl-Pi increased by 5.69–10.32%, while NaOH-Po decreased by 8.85–16.09% (Figure 2, Figure 3 and Figure S1).

Macroaggregates had the highest P content. Following the dryland-to-paddy conversion, NaOH-Pi exhibited the highest values in PF (with contents of 330.9 mg/kg in the silt+clay fraction, 381.9 mg/kg in the microaggregates, and 869.1 mg/kg in the macroaggregates), followed by Residual-P (with contents of 239.3, 209.3, and 429.3 mg/kg in the silt+clay, microaggregates and macroaggregates, respectively) (Figure 2). In contrast, H_2_O-Pi and conc. HCl-Po showed relatively low contents (Figure 2). Overall, the dryland-to-paddy conversion had the most pronounced impact on moderately active P (NaOH-Pi, NaOH-Po and HCl-Pi). Except for NaOH-Pi in the silt+clay fraction, the differences between DL and PF in these three P components were significant in the types of aggregates. (*p*< 0.05; Figure 2 and Figure 3 and Table S2).

### 3.2. Effects of Land-Use Change on Soil Phosphorus Cycling Functional Microorganisms

**Figure 4:**
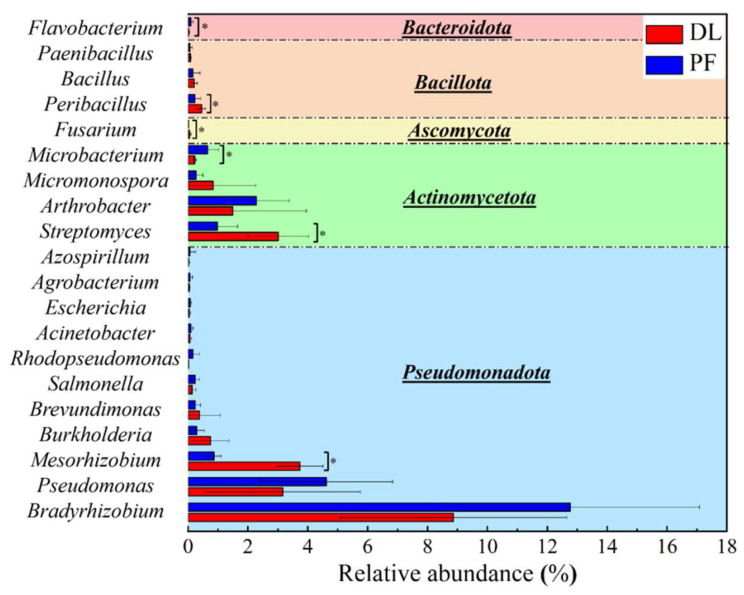
Relative abundance of the top 20 soil phosphorus cycling functional genera in dry-lands (DL)and paddy fields (PF). * indicate significant difference between DL and PF (*p <* 0.05).

**Figure 5:**
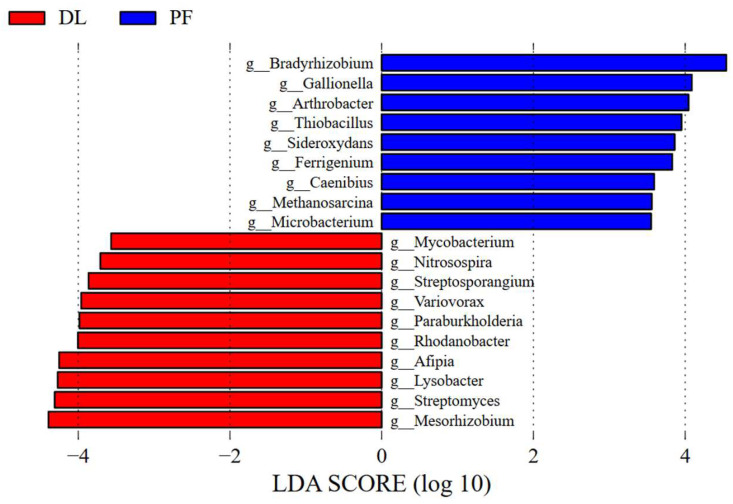
Bar plot of LDA scores from LEfSe analysis comparing dry-lands (DL)and paddy fields (PF)(LDA = 3.5).

Bacteria and fungi are the main components of the soil microorganisms in the study area. Among the top 20 genera involved in P cycling, 19 were bacteria and 1 was a fungus. Bacteria exhibited the highest relative abundance (58.32%) among soil microorganisms. At the phylum level, *Pseudomonadota*, *Actinomycetota* and *Bacillota* were the most represented phyla, with 11, four and three species identified, respectively. At the genus level, *Bradyrhizobium*, *Pseudomonas*, *Mesorhizobium*, *Streptomyces* and *Arthrobacter* exhibited the highest relative abundances, together accounting for 20.3% and 21.5% in DL and PF, respectively (Figure 4). After the conversion from dryland to paddy, soil microbial α-diversity exhibited a slight change without statistical significance (*p* > 0.05; Table S3), while a significant difference was observed in microbial β-diversity (*p* < 0.05; Table S4). Specifically, the relative abundances of *Streptomyces*, *Mesorhizobium*, *Peribacillus*, and *Fusarium* decreased significantly by 2.04%, 2.87%, 0.22%, and 0.04%, respectively, while those of *Flavobacterium* and *Microbacterium* increased significantly by 0.06% and 0.43% (*p* < 0.05; Figure 4). Additionally, the relative abundances of *Bradyrhizobium*, *Pseudomonas*, and *Arthrobacter* increased by 3.90%, 1.45%, and 0.79%, respectively, whereas the abundance of *Micromonospora* decreased by 0.55% (*p* > 0.05; Figure 4). Among these, *Bradyrhizobium* exhibited the largest change, increasing from 8.86% to 12.77%. LEfSe analysis further revealed the differential taxa between DL and PF (Figure 5). Among the PSMs, *Mesorhizobium* and *Streptomyces* were identified as the characteristic taxa for DL, while *Bradyrhizobium*, *Arthrobacter*, and *Microbacterium* were identified as the characteristic taxa for PF. These findings are consistent with the results shown in Figure 4.

### 3.3. Effects of Land-Use Change on Functional Genes Involved Soil Phosphorus Cycling

**Figure 6:**
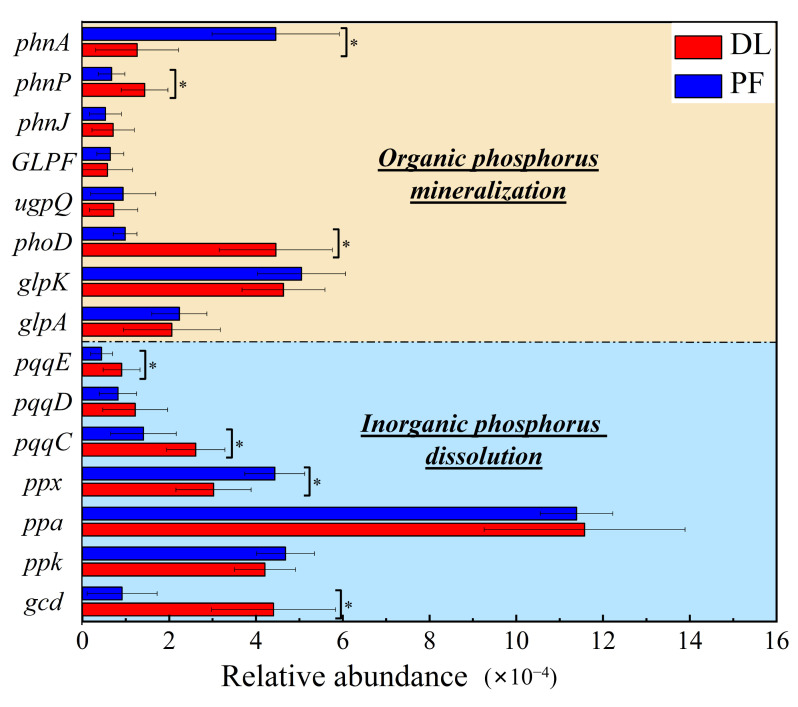
Relative abundance of phosphorus-cycling functional genes in dry-lands (DL)and paddy fields (PF). * indicate significant difference between DL and PF (*p <* 0.05).

For the DL and PF, the relative abundances of organic P mineralization genes were 0.18% and 0.17%, respectively, while those of inorganic P dissolution genes were 0.28% and 0.24% respectively (Figure 6). Regarding the organic P mineralization genes, *phnA*, *phoD*, *glpK* and *glpA* showed relatively higher abundances (Figure 6). As for the inorganic P dissolution genes, *pqqC*, *ppx*, *ppa*, *ppk* and *gcd* had relatively higher abundances (Figure 6). Moreover, the relative abundances of the *phnA*, *pqqE*, *pqqC* and *ppx* genes increased significantly, whereas those of the *phnP*, *phoD* and *gcd* genes decreased significantly, indicating that these genes may play key roles in the soil P cycle during the dryland-to-paddy conversion (*p* < 0.05; Figure 6).

After the dryland-to-paddy conversion, the types of key functional genes involved in soil P cycling remained unchanged, but their abundances changed obviously. Specifically, the relative abundances of genes related to inorganic P dissolution (e.g., *pqqE*, *pqqC* and *gcd*) decreased significantly, while that of *ppx* increased significantly (*p* < 0.05; Figure 6). Moreover, among the genes related to organic P mineralization, the abundance of *phnA* increased significantly, whereas abundances of *phnP* and *phoD* decreased significantly (*p* < 0.05; Figure 6). The changes in *gcd*, *phoD* and *phnA* were particularly pronounced, changing by approximately 0.03%, compared to changes of about 0.01% for other genes (Figure 6).

## 4. Discussion

**Figure 7:**
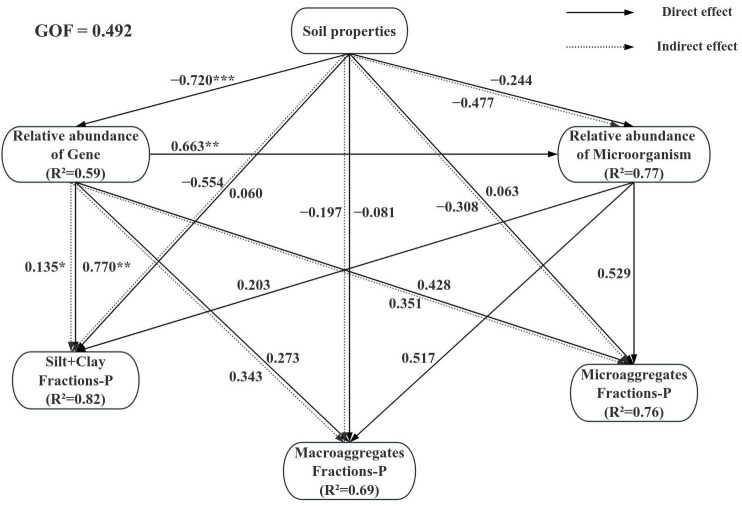
Structural equation model (SEM) analyzing relationships among basic soil properties, P-cycling functional microorganisms and genes, and aggregates-P fractions. The path coefficients represent the direction and magnitude of the effect of one variable on another. R^2^ indicates the proportion of variance explained by the model. Models fit was assessed using the goodness-of-Fit (GOF) statistic. For the model represented here, the GOF was 0.492. *, **, *** represent *p* < 0.05, 0.01, and 0.001, respectively.

According to the SEM results, significant causal relationships were identified among soil properties, the relative abundance of microorganisms and genes involved in P cycling, and P fractions in different aggregates. The model demonstrated a good fit with the data, as indicated by a goodness-of-fit (GOF) value of 0.492, and effectively illustrated both direct and indirect pathways among the variables (Figure 7 and Table S5).

Within the model, soil properties exerted a significant negative effect (−0.768) on the relative abundance of genes, and the relative abundance of genes showed a significant positive effect (0.797) on the P content of Silt+clay fraction (Figure 7). These results suggest that changes in basic soil properties significantly alter the expression of functional genes related to P solubilization, which in turn strongly influences the balance of P dynamics in the silt+clay fraction. This finding corroborates the work of Feng et al. [8], who demonstrated that changes in basic soil properties subsequently influence soil aggregates. Furthermore, the relative abundance of genes had a significant positive effect (0.532) on the relative abundance of microorganisms, indicating that the representation of functional genes is a key driver of the corresponding microbial community structure (Figure 7).

In summary, microbial composition and functional gene abundance play a vital role in the soil P cycle [17]. Changes in soil properties drive P transformation through their dual effects on the P fractions and microbial community structure.

### 4.1. Changes in Soil Basic Properties During the Dryland-to-Paddy Conversion: Dual Effects on P Fractions and Microbial Community Structure

Following the land-use conversion from DL to PF, the soil transitions from an aerobic environment to a long-term or intermittent anaerobic state, leading to a sharp decrease in redox potential [34]. The reduction of higher-valence Fe/Mn oxides consumes H^+^, resulting in a slight increase in pH (by 0.08 units), shifting the soil toward a less acidic condition (Table 1). Due to the buffering capacity of the soil, the pH fluctuates within a narrow range [35]. Moreover, rice straw return, combined with an anaerobic environment, slows the decomposition of SOM, significantly elevating the SOC content [36]. Furthermore, due to the changes in fertilization, irrigation and evaporation, the concentration of base ions in the soil increases, leading to a significant rise in the EC [37].

The changes in P species during the dryland-to-paddy conversion are closely related to the soil physicochemical properties. pH influences the precipitation and dissolution equilibria of phosphates, while EC affects their adsorption and desorption behavior. In acidic soils, a decrease in pH promotes P fixation by Fe^3+^ and Al^3+^, forming Fe/Al oxide precipitates, whereas an increase in pH enhances the availability of P [38]. Elevated EC increases the concentration of soluble salts (e.g., SO_4_^2−^, Cl^−^, Ca^2+^, K^+^), which compete with phosphate for adsorption sites on soil particles, promoting the desorption of fixed P [35,39]. High ionic strength in the soil solution can compress the diffuse double layer surrounding soil colloids [39]. This compression reduces the electrostatic potential at the colloid surface, thereby weakening the specific and non-specific adsorption of phosphate (PO_4_^3−^) onto variable-charge minerals and clay particle edges. As a result, more phosphate ions are released into the soil solution, increasing the bioavailable P [35,39]. Moreover, SOM decomposition products can form complexes with metal ions and compete with phosphate for adsorption sites on soil colloids, further reducing P fixation [40].

**Figure 8:**
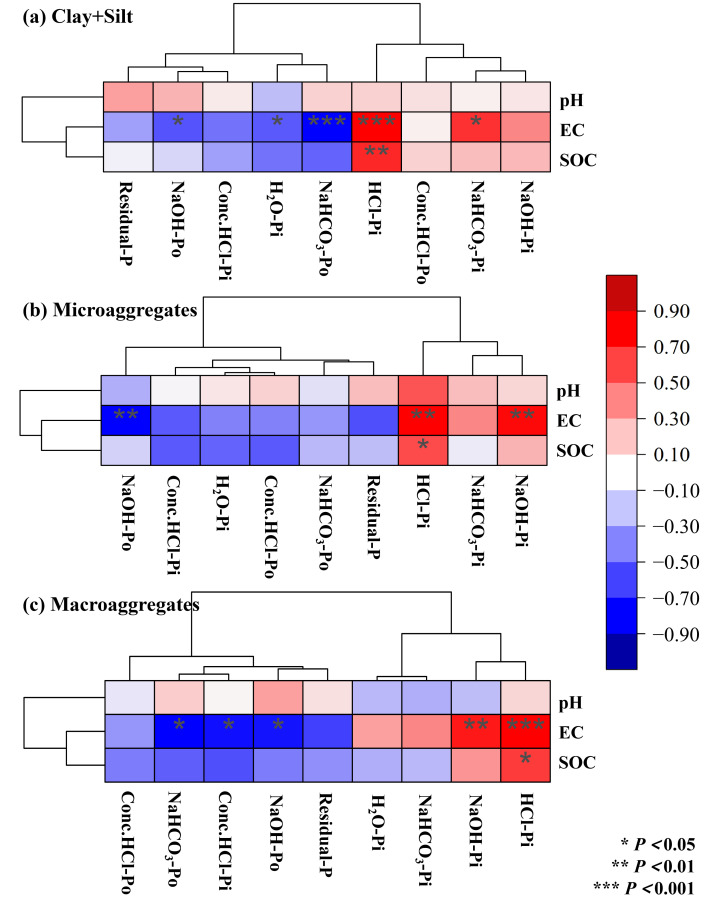
Clustering heatmap between pH, EC, SOC and P fractions in the three aggregate size classes.

In this study, EC showed a significant positive correlation with NaOH-Pi and HCl-Pi (*p* < 0.05; Figure 8). Conversely, EC had a negative correlation with highly active P, and a significant relationship occurred in the silt+clay fraction (*p* < 0.05; Figure 8). These findings indicate that EC plays a vital role in the transformation of P fractions from highly active P to moderately and lowly active P during the dryland-to-paddy conversion. The increase in SOC promoted the formation of microaggregates and macroaggregates, which increased by 130% and 355%, respectively (Table 1). These aggregates physically protect P and reduce its loss [5]. SOC showed a significant positive correlation with HCl-Pi, indicating its core role in the formation of lowly active P (*p* < 0.05; Figure 8). This study confirmed a 16.04% increase in the TP after dryland-to-paddy conversion, primarily attributable to macroaggregates (83.81%), whereas P in the silt+clay and microaggregates contributed only 11.08% and 5.11%, respectively (Figure 2). Furthermore, the application of inorganic P fertilizer significantly raises H_2_O-Pi, NaOH-Pi, Residual-P and NaHCO_3_-Pi, and slightly increases HCl-Pi, but has limited effects on organic P fractions [11,41]. This explains the prominent increases in lowly active HCl-Pi (64.68%) and moderately active NaOH-Pi (89.84%) in this study (Figure 2).

**Figure 9:**
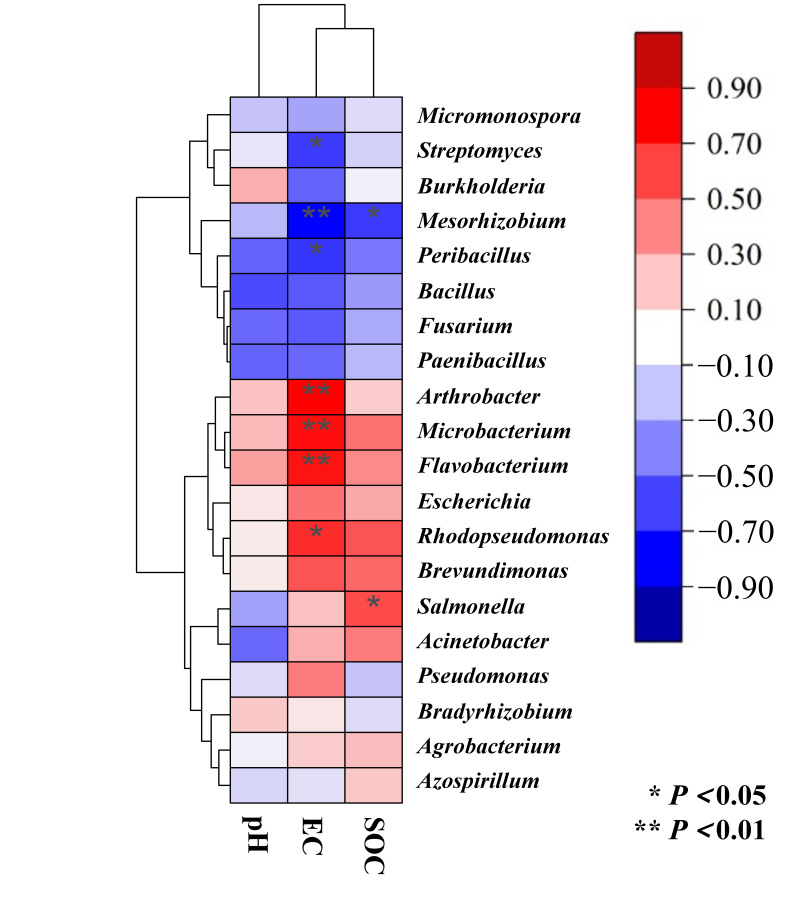
Clustering heatmap between pH, EC, SOC and the relative abundance of phosphorus-cycling functional genera.

The dryland-to-paddy conversion altered the structure and abundance of P cycling microorganisms, affecting the expression of key functional genes. After conversion, both EC and SOC increased significantly (*p* < 0.05; Table 1). As a core indicator of soil salinity, EC influences microbial biomass by regulating soil microbial respiration and enzyme activity, promoting microbial community succession [42,43]. P-cycling microorganisms engage in and mediate energy-dependent processes such as the secretion of organic acids and phosphatases [42]. The increase in SOC provides sources of carbon and energy, significantly enhancing microbial metabolic activity and altering community structure [44]. The correlation heatmap revealed that *Arthrobacter*, *Microbacterium*, *Flavobacterium*, *Rhodopseudomonas* and *Salmonella* were significantly positively correlated with EC and SOC, while *Streptomyces*, *Mesorhizobium* and *Peribacillus* were significantly negatively correlated with EC and SOC (*p* < 0.05; Figure 9). Therefore, changes in soil properties induced by dryland-to-paddy conversion play a crucial role in creating an environment favorable to microorganisms involved in the P cycle [45].

### 4.2. Changes in the Relative Abundances of Soil P Cycling Functional Genes and Microorganisms Dominated the Process of P Fraction Transformation

PSMs drive soil P cycling through the dissolution of inorganic P and the mineralization of organic P [46,47,48]. PSMs solubilize inorganic phosphates by secreting organic acids that lower the soil pH and chelate Fe^3+^ and Al^3+^ ions, thereby releasing fixed P and increasing P availability [49,50,51]. Moreover, PSMs catalyze the mineralization of organic P compounds (e.g., phytic acid, phospholipids, and nucleic acids) into plant-available inorganic phosphates, alleviating P limitation through enzymatic catalysis, environmental adaptation, and microbial interactions [16].

**Figure 10:**
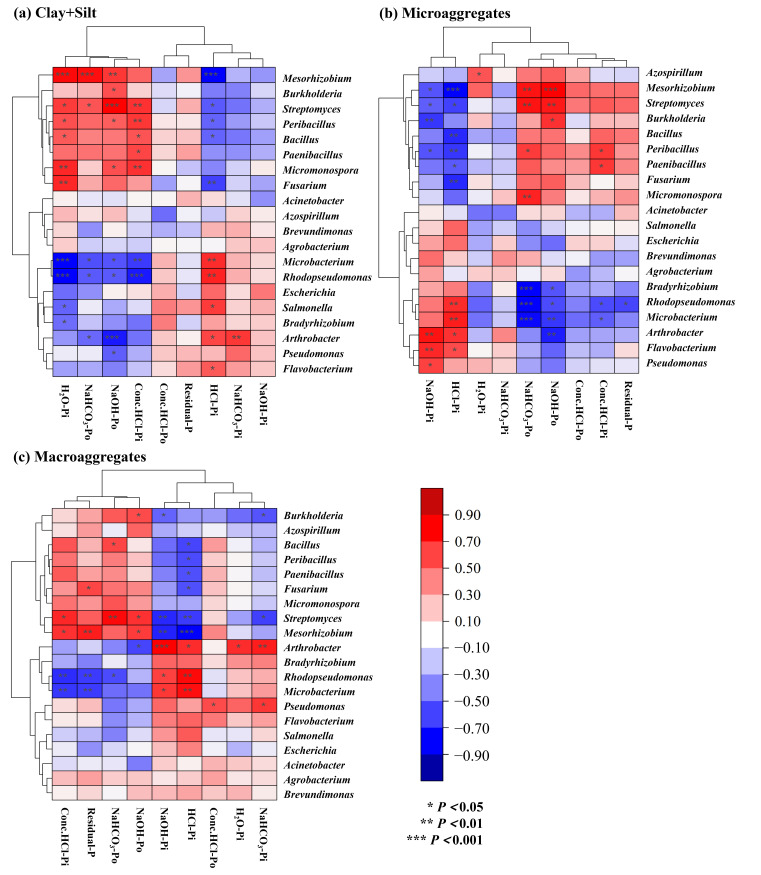
Clustering heatmap between P fractions in aggregates and the relative abundance of phosphorus-cycling functional genera.

The conversion from dryland to paddy induced distinct shifts in the microbial community, characterized by significant decreases in *Streptomyces*, *Mesorhizobium*, *Peribacillus* and *Fusarium*, and significant increases in *Flavobacterium* and *Microbacterium* (*p* < 0.05). The correlation heatmap revealed that *Bradyrhizobium* was significantly negatively correlated with NaOH-Po in microaggregates; *Pseudomonas* showed a significant positive correlation with NaOH-Po in the clay+silt fraction and a negative correlation with NaOH-Pi in microaggregates; *Streptomyces* was significantly positively correlated with NaOH-Po across all aggregates and negatively correlated with HCl-Pi and NaOH-Pi; and *Mesorhizobium* exhibited a significant positive correlation with NaOH-Po in microaggregates and a negative correlation with HCl-Pi in all aggregates (*p* < 0.05; Figure 10). Furthermore, *Mesorhizobium*, *Streptomyces*, *Peribacillus* and *Fusarium* showed synergistic interactions, as did *Microbacterium*, *Bradyrhizobium*, *Arthrobacter*, *Pseudomonas*, *Flavobacterium* (Figure 10).

Studies have confirmed that *Bradyrhizobium*, *Pseudomonas*, and *Mesorhizobium* possess both N fixation and P solubilization capabilities. Under high N conditions, these bacteria may preferentially utilize available environmental N, thereby suppressing N-fixation gene expression and reallocating energy allocation to P metabolism [52,53]. After the dryland-to-paddy conversion, the simultaneous increases in SOC, TN and TP may lead to *Bradyrhizobium* enhancing P metabolism, reducing the accumulation of lowly active P, while *Pseudomonas* and *Mesorhizobium* weaken P metabolism, thereby promoting the accumulation of lowly active P [54]. Moreover, *Streptomyces* can produce various extracellular enzymes to decompose recalcitrant organic residues and release N and P [55]. The phosphate ions released during the decomposition of SOM are prone to combine with metal ions such as Ca, Fe and Al, thereby affecting the moderately active P dynamics in the soil [55,56]. After the dryland-to-paddy conversion, the abundance of *Bradyrhizobium* and *Pseudomonas* increased obviously, while that of *Streptomyces* and *Mesorhizobium* decreased significantly (Figure 4). These compositional shifts in microbial communities primarily drove the increase in moderately and lowly active P within the silt+clay fraction and macroaggregates, and the decrease within the microaggregates.

**Figure 11:**
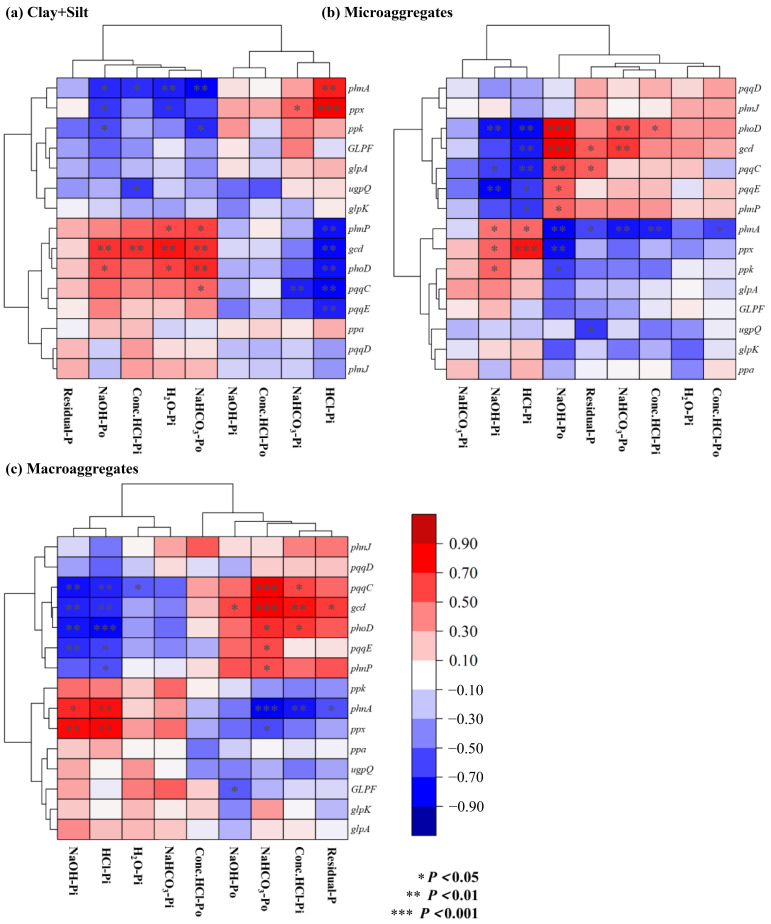
Clustering heatmap between aggregates-P fractions and relative abundance of phosphorus cycling functional genes.

While the conversion from dryland to paddy did not alter the types of key functional genes involved in soil P cycling, it significantly reshaped their abundance profiles, with genes governing inorganic P dissolution and organic P mineralization exhibiting distinct and pronounced shifts (Figure 11). *gcd* and *phoD* showed a significant positive correlation with NaOH-Po and a negative correlation with NaOH-Pi and HCl-Pi across all three aggregates (*p* < 0.05; Figure 11). Conversely, *phnA* was significantly positively correlated with NaOH-Pi and HCl-Pi, but negatively correlated with NaOH-Po (*p* < 0.05; Figure 11). Furthermore, *phnA* and *ppx* showed synergistic interactions, as did *pqqE*, *pqqC*, *gcd*, *phnP* and *phoD* (Figure 11).

In summary, the key genes affecting the transformation of moderately active P following the land-use conversion were *gcd*, *pqqC*, *pqqE*, *phnP*, *phoD*, *ppx* and *phnA*. The decrease in the abundance of *gcd*, *pqqC*, *pqqE*, *phnP* and *phoD*, coupled with an increase in the abundance of *ppx* and *phnA*, led to a reduction in moderately active P in microaggregates and macroaggregates, while in the silt+clay fraction the effect primarily manifested as a decrease in inorganic P. Changes in the abundance of these key genes influence soil P cycling microbial communities and drive the transformation of soil P fractions.

## 5. Conclusions

This study investigated the effects of dryland-to-paddy conversion on the soil aggregate P fractions and microbial functional diversity in a typical black soil region. The results demonstrated that the proportion of microaggregates and macroaggregates increased significantly during the conversion from DL to PF. The increase in macroaggregates-P contributed to 83.81% of the increase in TP. The dominant P fraction was shifted from NaOH-Po to NaOH-Pi and HCl-Pi during the conversion. Increases in *Pseudomonas* and *Bradyrhizobium*, along with decreases in *Strepromyces* and *Mesorhizobium,* drove the transformation of P fractions. The key functional genes regulating P transformation were *gcd*, *phoD* and *phnA*. These findings suggest that alterations in basic soil properties following dryland-to-paddy conversion act as the underlying drivers, influencing aggregate P fractions and in turn shaping both microbial community structure and the abundance of key P-cycling functional genes. However, this study did not capture the temporal dynamics of P forms and microbial community structure across different stages of land-use conversion. Future research should explore the dynamics of P fractions, microbial communities, and functional genes throughout the dryland-to-paddy conversion process to better elucidate the evolutionary mechanism of P transformation. This study provides important insights for evaluating P management strategies following the dryland-to-paddy conversion.

## Figures and Tables

**Table 1:** Basic soil properties in dry-lands and paddy fields.

Land Use		DL ^†^	PF
pH	(-)	5.75 ± 0.23 a ^‡^	5.83 ± 0.39 a
EC	(ms/m)	6.08 ± 1.42 b	9.90 ± 2.60 a
SOC	(g/kg)	22.60 ± 4.11 b	26.48 ± 7.34 a
TN		1.92 ± 0.28 a	2.16 ± 0.58 a
TP		0.85 ± 0.16 a	0.99 ± 0.26 a
silt+clay	(%)	86.51 ± 18.11 a	58.28 ± 17.39 b
Microaggregates		11.08 ± 13.40 b	25.49 ± 9.50 a
Macroaggregates		2.41 ± 4.85 b	10.97 ± 16.23 a

^†^ DL, dry-lands; PF, paddy fields. ^‡^ All values are presented as means ± SD. Significant differences between DL and PF are denoted by different lowercase letters (*p* < 0.05).

## Data Availability

The original contributions presented in this study are included in the article/Supplementary Material. Further inquiries can be directed to the corresponding authors.

## Supplementary Materials

The following supporting information can be downloaded at: https://www.mdpi.com/article/10.3390/microorganisms14030658/s1, Figure S1: Proportion of P fraction in soil aggregates at dry-lands and paddy fields; Table S1: Nutrient classification standards from the second national soil survey (g/kg); Table S2: Content of P fractions in soil aggregates in dry-lands and paddy fields; Table S3: Soil α-diversity in dry-lands and paddy fields; Table S4: Soil β-diversity in dry-lands and paddy fields; Table S5: Summary data of PLS-PM path model.
