# Supplementary material for: Effect of Dryland-to-Paddy Conversion on Soil Aggregate Phosphorus Fractions and Microbial Functional Diversity in a Typical Black Soil Region of the Sanjiang Plain
Source: Microorganisms. 2026 Mar 14;14(3):658. doi: 10.3390/microorganisms14030658 (PMC13029029; doi:10.3390/microorganisms14030658)
Supplement: Supplementary file 1 [file microorganisms-14-00658-s001.zip › microorganisms-4145610-supplementary.pdf]

Supplementary Material

for

Effect of dryland-to-paddy conversion on soil aggregate phosphorus fractions and microbial functional diversity in a typical black soil region of the Sanjiang Plain

Contents

Figure S1. Proportion of P fraction in soil aggregates at dry-lands and paddy fields;

Table S1. Nutrient classification standards from the second national soil survey (g/kg);

Table S2. Content of P fractions in soil aggregates in dry-lands and paddy fields;

Table S3. Soil α-diversity in dry-lands and paddy fields;

Table S4. Soil β-diversity in dry-lands and paddy fields;

Table S5. Summary data of PLS-PM path model.

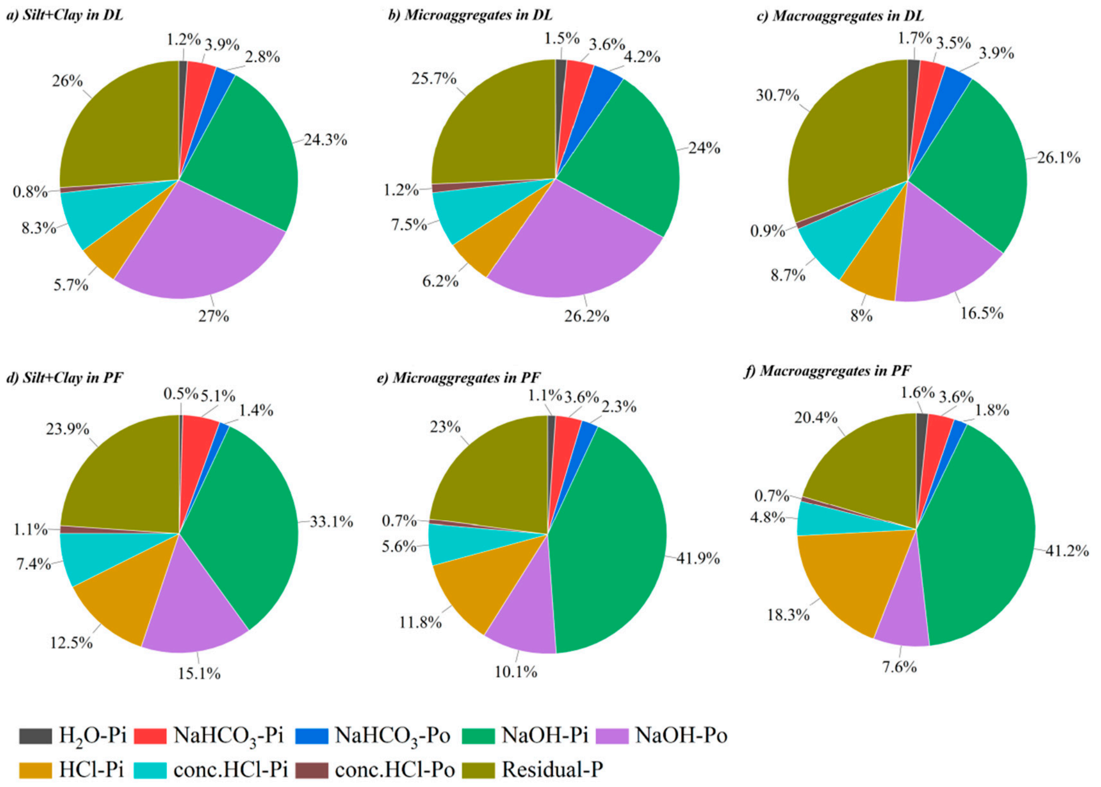

Figure S1. Proportion of P fraction in soil aggregates at dry-lands and paddy fields.

Table S1. Nutrient classification standards from the second national soil survey (g/kg)[1].

**Grade**	**Ⅰ -Extremely rich**	**Ⅱ -Rich**	**Ⅲ-Relatively rich**	**Ⅳ -Moderate**	**Ⅴ-Poor**	**Ⅵ-Extremely poor**
TP (g/kg)	＞1	0.8-1	0.6-0.8	0.4-0.6	0.2-0.4	＜0.2

Table S2. Content of P fractions in soil aggregates in dry-lands and paddy fields.

P Fractions	Soil aggregates	DL† mg/kg	PF mg/kg
H_2O-Pi	clay+silt	11.27±5.25a‡	4.83±1.26b
	Microaggregates	12.22±4.42a	9.84±4.64a
	Macroaggregates	27.88±14.91a	34.01±12.96a
$NaHCO_3$-Pi	clay+silt	38.08±6.83a	50.67±20.11a
	Microaggregates	30.31±11.40a	32.81±8.40a
	Macroaggregates	56.84±22.08a	75.59±24.68a
$NaHCO_3$-Po	clay+silt	26.93±10.95a	14.10±6.69a
	Microaggregates	35.28±11.40a	20.71±7.10b
	Macroaggregates	63.9±23.20a	38.78±29.46a
NaOH-Pi	clay+silt	235.11±48.05a	330.89±168.87a
	Microaggregates	201.78±55.30b	381.89±141.19a
	Macroaggregates	425.22±148.42b	869.11±347.16a
NaOH-Po	clay+silt	261.44±70.12a	151.44±66.48b
	Microaggregates	220.44±70.76a	92.14±44.91b
	Macroaggregates	268.22±116.52a	160.86±82.82b
HCl-Pi	clay+silt	54.98±11.73b	124.82±38.22a
	Microaggregates	51.77±11.50b	107.93±23.40a
	Macroaggregates	130.31±19.81b	386.56±103.37a
conc. HCl-Pi	clay+silt	80.16±11.24a	73.64±41.84a
	Microaggregates	63.11±17.44a	50.69±19.92a
	Macroaggregates	141.64±38.90a	100.97±48.65a
conc. HCl-Po	clay+silt	7.41±5.15a	10.73±5.14a
	Microaggregates	10.19±3.44a	6.28±3.73b
	Macroaggregates	15.02±5.45a	14.87±7.61a
Residual-P	clay+silt	251.89±71.39a	239.33±86.56a
	Microaggregates	216.33±18.77a	209.33±85.28a
	Macroaggregates	499.22±69.05a	429.33±100.49a

† DL, dry-lands; PF, paddy fields.

‡ All values are presented as means±SD. Significant differences between DL and PF are denoted by different lowercase letters (P< 0.05).

Table S3. Soil α-diversity in dry-lands and paddy fields.

Land use	DL†	PF
Chao1 Index	1921±262a‡	1850±425a
Shannon Index	5.91±0.37a	5.87±0.39a
Simpson Index	0.89±0.03a	0.89±0.03a

† DL, dry-lands; PF, paddy fields.

‡ All values are presented as means±SD. Significant differences between DL and PF are denoted by different lowercase letters (P< 0.05).

Table S4. Soil β-diversity in dry-lands and paddy fields.

	Land use	Distances to DL†	Distances to PF
Anosim Index	DL	0.40±0.06b‡	0.48±0.06a
	PF	0.48±0.06a	0.37±0.05b
Permanova Index	DL	0.58±0.02b	0.67±0.03a
	PF	0.67±0.03a	0.61±0.03b

† DL, dry-lands; PF, paddy fields.

‡ All values are presented as means±SD. Significant differences between DL and PF are denoted by different lowercase letters (P< 0.05).

Table S5. Summary data of PLS-PM path model.

			Direct effect	Indirect effect	Total effect
Soil properties	→	Relative abundance of Gene	-0.768		-0.768
Soil properties	→	Relative abundance of Microorganism	-0.398	-0.409	-0.807
Soil properties	→	Silt+Clay Fractions-P	0.126	-0.612	-0.486
Soil properties	→	Microaggregates Fractions-P	0.085	-0.314	-0.229
Soil properties	→	Macroaggregates Fractions-P	-0.092	-0.214	-0.306
Relative abundance of Gene	→	Relative abundance of Microorganism	0.532		0.532
Relative abundance of Gene	→	Silt+Clay Fractions-P	0.797	0.127	0.924
Relative abundance of Gene	→	Microaggregates Fractions-P	0.409	0.303	0.712
Relative abundance of Gene	→	Macroaggregates Fractions-P	0.279	0.266	0.545
Relative abundance of Microorganism	→	Silt+Clay Fractions-P	0.239		0.239
Relative abundance of Microorganism	→	Microaggregates Fractions-P	0.570		0.570
Relative abundance of Microorganism	→	Macroaggregates Fractions-P	0.500		0.500

References

[1]National Soil Survey Center. Soils in China; China Agriculture Press: Beijing, China, 1998.
